# Fit-for-purpose biomarker method validation for application in clinical trials of anticancer drugs

**DOI:** 10.1038/sj.bjc.6605910

**Published:** 2010-10-05

**Authors:** J Cummings, F Raynaud, L Jones, R Sugar, C Dive

**Affiliations:** 1 Clinical and Experimental Pharmacology, Paterson Institute for Cancer Research, University of Manchester, Wilmslow Road, Manchester M20 4BX, England; 2 Cancer Research UK Centre for Cancer Therapeutics, The Institute for Cancer Research, Haddow Laboratories, 15 Cotswold Road, Sutton, Surrey SM2 5N, UK; 3 Translational Research Team, Clinical and Translational Operations and Funding Directorate, Cancer Research UK, 61 Lincolns Inn Fields, London WC2A 3PX, UK; 4 The Drug Development Office, Clinical and Translational Operations and Funding Directorate, Cancer Research UK, 61 Lincolns Inn Fields, London WC2A 3PX, UK

**Keywords:** biomarkers, validation, clinical trials, fit-for-purpose

## Abstract

Clinical development of new anticancer drugs can be compromised by a lack of qualified biomarkers. An indispensable component to successful biomarker qualification is assay validation, which is also a regulatory requirement. In order to foster flexible yet rigorous biomarker method validation, the fit-for-purpose approach has recently been developed. This minireview focuses on many of the basic issues surrounding validation of biomarker assays utilised in clinical trials. It also provides an overview on strategies to validate each of the five categories that define the majority of biomarker assays.

During anticancer drug development, biomarkers can have many applications. They may be utilised as discovery tools, as pharmacodynamic (PD) markers of drug mechanism or efficacy both preclinically and in early phase trials, and as predictive indices of patient response in late phase trials (Lee *et al*, 2005; Sarker and Workman, 2007). Thus, incorporation of biomarkers into clinical trials has the potential to guide and accelerate the pace of development of new anticancer drugs (McShane *et al*, 2009). Biomarkers may also provide a diagnostic readout on tumour biology in experimental cancer medicine or be prognostic or predictive of disease or therapeutic outcome (Ludwig and Weinstein, 2005). However, at present there are few fully qualified biomarkers available to utilise in clinical trials of anticancer drugs (Jain *et al*, 2009). Assay characterisation remains a critical component in biomarker qualification and often biomarkers can fail not because of the underlying science, but because of poor choice of assay and lack of validation (Carden *et al*, 2010).

The UK clinical trials regulations state that ‘systems with procedures that assure the quality of every aspect of the trial should be implemented’, which includes method validation (Cummings *et al*, 2008). Although there is now a raft of regulatory guidance documents associated with clinical trials these make few references to method validation and there is a need for amplification on many of the issues associated with biomarker analysis (Cummings *et al*, 2008). Recently the ‘fit-for-purpose’ approach has emerged to provide guidance on biomarker method validation (Lee *et al*, 2005, 2006; Tan *et al*, 2009). The present commentary seeks to clarify and extend this approach.

## Fit-for-purpose biomarker method validation

The benchmark definition of method validation has been presented by the International Organisation for Standardisation as ‘the confirmation by examination and the provision of objective evidence that the particular requirements for a specific intended use are fulfilled’. Although this definition appears self evident, its full implications are often overlooked (Feinberg *et al*, 2004; Boulanger *et al*, 2009). Thus, ideally validation should progress down two parallel tracks which eventually converge—one experimental, the other operational. The first is to establish the purpose of the method and agree upon outcomes, target values or acceptance limits, whereas the second is to characterise the performance of the assay by experimentation. The critical step in the whole process then rests on the evaluation of technical performance against the predefined purpose. If the assay can deliver to expectations, then it is deemed fit for that purpose. If not, then it cannot be considered fit for the specified purpose. Comparing performance specifications against predefined acceptance limits in isolation of purpose does not conform to the strict definition of method validation. Nevertheless, technical data can have an important role in verifying that the assay is working properly and in aiding in the diagnosis of faults (Rozet *et al*, 2007).

**Figure 1:**
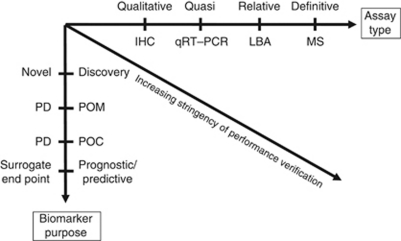
Overview of fit-for-purpose biomarker method validation. The fit-for-purpose approach to biomarker method validation tailors the burden of proof required to validate an assay to take account of both the nature of technology utilised and position of the biomarker in the spectrum between research tool and clinical end point. Ultimately, fit-for-purpose requires an assessment of the technical ability of the assay to deliver against the predefined purpose. Abbreviations: IHC=immunohistochemistry; LBA=ligand binding assay; MS=mass spectrometry; PD=pharmacodynamic; POM=proof of mechanism; POC=proof of concept.

The position of the biomarker in the spectrum between research tool and clinical end point dictates the stringency of experimental proof required to achieve method validation (Lee *et al*, 2007). In addition, the nature of the analytical technology also influences the level of performance verification required (Figure 1). The availability of novel technology platforms such as the omics technologies allow more scientific questions to be posed, but represent additional analytical challenges in terms of assay validation. In many cases, these technologies will provide a set of biomarker candidates that need to be verified by a more robust and conventional analytical method.

## How to conduct fit-for-purpose biomarker method validation?

The American Association of Pharmaceutical Scientists (AAPS) and the US Clinical Ligand Society have identified five general classes of biomarker assays (Lee *et al*, 2005). A definitive quantitative assay makes use of calibrators and a regression model to calculate absolute quantitative values for unknowns. The reference standard is fully characterised and representative of the biomarker. A relative quantitative assay uses a response–concentration calibration with reference standards that are not fully representative of the biomarker. A quasi-quantitative assay does not employ a calibration standard, but has a continuous response that can be expressed in terms of a characteristic of the test sample. Qualitative (categorical) assays can either be described as ordinal reliant on discrete scoring scales like those used in immunohistochemistry (IHC) or nominal that pertains to a yes/no situation; for example, the presence or absence of a gene product (Lee *et al*, 2005, 2006, 2007). Table 1 represents the consensus position on which parameters should be investigated for each class of biomarker assay.

## The different phases of fit-for-purpose biomarker assay validation

Biomarker method validation can be envisaged as proceeding through discrete stages (Shah *et al*, 2000; Lee *et al*, 2005, 2006). The first stage is where definition of purpose and selection of the candidate assay occurs, and is perhaps the most critical. During stage 2 the goal is to assemble all the appropriate reagents and components, write the method validation plan and decide upon the final classification of the assay. Stage 3 is the experimental phase of performance verification leading to the all important evaluation of fitness-for-purpose culminating in writing a standard operating procedure. In-study validation (stage 4) allows further assessment of fitness-of-purpose and the robustness of the assay in the clinical context and enables identification of patient sampling issues, such as collection, storage and stability. Stage 5 is where the assay enters routine use, and here, quality control (QC) monitoring, proficiency testing and batch-to-batch QC issues can be fully explored. The driver of the process is one of continual improvement, which may necessitate a series of iterations that can lead back to any one of the earlier stages (Lee *et al*, 2006, 2007).

## Validation of definitive quantitative biomarker assays

Examples of definitive quantitative biomarker methods include mass spectrometric analysis. The objective of a definitive quantitative method is to determine as accurately as possible the unknown concentrations of the biomarker in the patient samples under investigation (Rozet *et al*, 2007). Analytical accuracy is dependant on the total error in the method, consisting of the sum of the systematic error component (bias) and the random error component (intermediate precision; DeSilva *et al*, 2003). Total error takes account of all relevant sources of variation: for example, day, analyst, analytical platform or batch (Cummings *et al*, 2008).

Recognised performance standards have been established for bioanalysis of small molecules by the pharmaceutical industry (Shah *et al*, 1991, 2000). An evaluation of both precision (% coefficient of variation (CV)) and accuracy (mean % deviation from nominal concentration) is required. Repeat analyses of the pre-study validation samples (VS) are expected to vary by <15%, except at the lower limit of quantitation (LLOQ) where 20% is acceptable. During in-study patient sample analysis, quality control samples (QCs) should be employed at three different concentrations spanning the calibration curve. A run is accepted as valid when at least 67% (4/6) of the QCs fall within 15% of their nominal values (the 4 : 6 : 15 rule; Shah *et al*, 1991, 2000).

More flexibility is allowed in biomarker method validation where during pre-study validation each assay can be evaluated on a case per case basis, with 25% being the default value (30% at the LLOQ) for precision and accuracy. A similar approach may be adopted during patient sample analysis in setting acceptance limits for QCs, either in terms of a 4 : 6: X rule or through adoption of confidence intervals (Lee *et al*, 2005, 2006, 2007). On a note of caution, applying fixed performance criteria in the absence of statistical evaluation that they are relevant to the assay under investigation has been challenged (Findlay, 2008, 2009). Adopting a 4 : 6 : X rule of acceptability for the QCs could mean at best that 33% of the patient samples will also not fall within the acceptance limits. Researchers have seriously questioned whether a method can be considered fit-for-purpose on the basis of a 4 : 6 : X rule (Boulanger *et al*, 2009). The Societe Francaise des Sciences et Techniques Pharmaceutiques (SFSTP) has published a series of papers on fit-for-purpose validation of quantitative analytical techniques based on an ‘accuracy profile’ (Boulanger *et al*, 2003; Feinberg *et al*, 2004). The accuracy profile takes account of total error (bias and intermediate precision), a pre-set acceptance limit that the user defines and produces a plot based on the ‘*β*-expectation tolerance interval’ that displays the confidence interval (e.g. 95%) for future measurements. Effectively, the accuracy profile allows researchers to visually check what percentage of future values are likely to fall with the pre-defined acceptance limit. To construct an accuracy profile the SFSTP recommend that 3–5 different concentrations of calibration standards and 3 different concentrations of VS (representing high, medium and low points on the calibration curve) are run in triplicate on 3 separate days, (Feinberg *et al*, 2004; Feinberg, 2007; Rozet *et al*, 2007). Biomarkers methods may require a greater number of calibration standards and VS due to nonlinearity.

Other performance parameters such as sensitivity, dynamic range, LLOQ and upper limit of quantitation (ULOQ) can be obtained from the accuracy profile. As with all five categories of biomarker assays sample and reagent integrity should also be carefully assessed during method validation, including studies on sample stability during collection, storage and analysis (Nowatzke and Wood, 2007). Studies on specificity, dilution linearity and parallelism with a definitive quantitative biomarker are essential, but are perhaps less problematic than with a relative quantitative assay (see below), as the VS are by their nature more similar in composition to patient samples and should behave in a similar manner.

## Validation of relative quantitative biomarker assays

The ligand binding assay (LBA) is the archetypical quantitative assay for endogenous (protein) macromolecular biomarkers. However, as most biomarker LBA ligands are endogenous substances and already present in patient/volunteer samples, an analyte-free matrix to utilise during validation studies is more difficult to obtain. Access to a fully characterised form of biomarker to act as a calibration standard is also limited. Thus, most available biomarker LBA fall into the category of relative quantitation (Lee *et al*, 2005).

Ligand binding assay is associated with a multiplicity of specificity issues (Findlay, 2008). Biotransformation caused by a variety of factors can introduce new forms of the biomarker into samples with ill-defined behaviour in the ELISA assay (Mahler *et al*, 2009). Complexation of the ligand with a soluble receptor, protein aggregation, folding/unfolding of the ligand can mask or reveal antibody epitope binding sites (Cummings *et al*, 2007). Ligand binding assay are also dependant on the integrity of reagents such as antibodies, which are subject to their own issues of supply and stability. Concentrations of the biomarker in the disease group of interest are often unknown, and thus, target expectations are more difficult to define in advance. Ligand binding assay are also susceptible to sample non-dilution linearity and interference with heterophilic antibodies can result in false positive results (Findlay, 2009).

Only precision and bias can be evaluated during pre-study validation, not accuracy. Nonetheless, depending on the nature of the calibration standards and matrix of choice, precision and bias determined in VS and QCs may reflect only poorly the true analytical behaviour of the assay with patient samples. As the calibration curve for most LBA are non-linear, the AAPS recommend that at least 8–10 different non-zero concentration should be run on 3–6 separate occasions to establish the most appropriate calibration model (DeSilva *et al*, 2003). Careful attention should be paid to the curve fitting routine such as 4 or 5-PL and to weighting, and the working effective range of the assay should be based on the precision profile where the deviation from the line of best fit for back calculated values should lie within an acceptance limit of 10–20% (Kelley and DeSilva, 2007). During pre-study validation, at least five different concentrations of VS spanning the calibration curve—including LLOQ; 3 × LLOQ, mid-range, high and ULOQ—should be analysed in duplicate on at least six different runs. Inter-batch precision and bias should be <±20% for each parameter, except at the LLOQ and the ULOQ where ±25% is acceptable (Viswanathan *et al*, 2007). Similar acceptance limits were recommended for in-study validation with QCs, but here only three different concentrations were required to be run in duplicate and a 4 : 6 : X rule utilised. These recommendations have been largely adopted in biomarker method validation, but with allowances to extend acceptance criteria if scientifically justified (Lee *et al*, 2005, 2006, 2007). However, we believe that it is preferable that acceptance criteria should be based on total error coupled to a confidence interval of 95%, especially with the higher degree of uncertainty associated with relative quantitative methods (Lee *et al*, 2005, 2006, 2007). For reasons stated above, the 4 : 6 : X rule should be avoided.

Specificity is defined as the ability to measure the analyte of interest in the presence of other components in the assay matrix. There are two types of non-specificities: specific non-specificity and non-specific non-specificity (Kelley and DeSilva, 2007). Specific non-specificity can result in interference from macromolecules structurally related to or derived from the biomarker. Non-specific non-specificity (matrix effect) can result in interference from unrelated species and matrix components, but can often be eliminated by dilution of sample in an appropriate buffer. To prove specificity, the AAPS requires evaluation of the concentration–response relationships of both spiked and non-spiked samples obtained from 6–10 different patient derived sources.

Recently, incurred (patient) sample reanalysis (ISR) has been strongly recommended in bioanalysis as a more rigorous test of assay reproducibility, rather than the use of QCs (Fast *et al*, 2009). Such an approach has even greater relevance in all five categories of biomarker assays and especially *in situations* where QCs are usually less representative of clinical samples, such as in the case of relative quantitative techniques (Findlay, 2009).

The study of dilution linearity and especially parallelism in performance verification of relative quantitative assays such as LBA cannot be over emphasised. Dilution linearity is normally studied with spiked QCs during pre-study method validation and care has to be exercised in the choice of matrix to act as the diluent (Greystoke *et al*, 2008). Whereas, parallelism is assessed using multiple dilutions of study samples that fall on the quantitative range of the calibration curve and can be conducted on either individual patient samples or with a pool of patient samples (Kelley and DeSilva, 2007). There are two ways of representing parallelism: the first is as a plot of measured concentrations for the patient samples against 1/dilution factor using log scales. A linear regression analysis is performed and acceptance criteria can be based either on correlation coefficients or a statistical acceptance criterion of <20% for the deviation of each dilution from the line of best fit (Findlay *et al*, 2000). Alternatively, a plot of measured concentrations for the patient samples x dilution factor against 1/dilution can be constructed. This should yield a flat line so that the CV among the recovered concentrations at different dilutions can be used to verify parallelism and here a CV of <30% is reported as the acceptance limit (Kelley and DeSilva, 2007).

## Validation of quasi-quantitative biomarker assays

This category of biomarker assay lacks calibration against a certified standard, but reports numerical values as a characteristic of the sample. Examples of such assays include quantitative RT–PCR. Performance parameters that form the core of pre-study validation include precision, specificity, sensitivity and the dynamic range of the assay.

To illustrate the principles of fit-for-purpose validation of a quasi-quantitative assay an ELISA measuring DNA nucleosomes (nDNA) as a biomarker of cell death (Cell Death Detection ELISA^Plus^ from ROCHE Diagnostics, Ltd., Burgess Hill, UK) is chosen as a typical example. This particular sandwich ELISA is only supplied with a positive control and is not calibrated against a standard, so assay readout is in absorbance units generated by the microplate reader. In addition, QCs are prepared with the positive control, but are of limited value, as they are reconstituted in buffer. The fit-for-purpose approach adopted therefore focused on demonstrating utility in the analysis of clinical trial samples. Stability studies focussed on nDNA spiked into serum or plasma to replicate patient samples. Careful handling of whole blood was critical in avoiding haemolysis, while isolation of serum or plasma was essential as soon as practicable in order to store samples at −80 °C, before analysis. A protocol was developed for assay validation during in study analysis of patient samples that included 4–6 replicates of the positive control QC to verify assay performance and up to 6 patient samples for ISR, as the primary test of QC and reproducibility. Application of the assay to the analysis of clinical specimens confirmed clinical utility both as a PD marker (Dean *et al*, 2009) and as a predictive biomarker of response to cancer chemotherapy (Hou *et al*, 2009). Paradoxically, biomarker assays such as quasi-quantitative or qualitative techniques that require verification of fewer performance characteristics, have perhaps an even greater requirement to be fast tracked into the clinic in order to complete or even initiate the process of validation.

## Validation of qualitative biomarker assays

Often positioned at the diagnostic end of the biomarker spectrum, examples of qualitative biomarker assays include western blotting, IHC and fluorescence *in situ* hybridisation. Positive and negative controls are the mainstay to confirm assay performance (Lee *et al*, 2005, 2006). Proving analytical and more importantly clinical specificity and sensitivity is the key to validation of this category of biomarker assay, but such a process can require access to resources which are not readily available, such as knock out mice, cell lines with overexpression of the target protein or clinical specimens. Under these circumstances, a risk-based approach to proving specificity is recommended, where more limited studies are conducted with resources that are more readily available. The Clinical Laboratory Standards Institute (CLSI) evaluation protocol for a qualitative diagnostic test recommends analysis of a minimum of 50 positive and 50 negative specimens run over 10–20 days, in the absence of an existing body of validation data. If, however, a body of validation can be provided by the vendor or supplier, then more limited studies to confirm performance within manufacturer's specifications should be adequate.

In conclusion, the realisation that patients have consented in good faith to donate samples for clinical research mandates the scientific community to ensure that experimental design is appropriate and that the assays employed are validated. Fit-for-purpose validation provides biomarker researchers with an approach that tailors the burden and stringency of proof required to validate an assay to take account of both the nature of technology utilised and the context in which the biomarker will be applied.

## Figures and Tables

**Table 1:** Recommended performance parameters that should be evaluated during biomarker method validation based on assay technology category

**Performance characteristic**	**Definitive quantitative**	**Relative quantitative**	**Quasi-quantitative**	**Qualitative**
Accuracy	+			
Trueness (bias)	+	+		
Precision	+	+	+	
Reproducibility				+
Sensitivity	+	+	+	+
	LLOQ	LLOQ		
Specificity	+	+	+	+
Dilution linearity	+	+		
Parallelism	+	+		
Assay range	+	+	+	
	LLOQ–ULOQ	LLOQ–ULOQ		

Abbreviations: LLOQ=lower limit of quantitation; ULOQ=upper limit of quantitation.

## References

[bib1] Boulanger B, Chiap P, Dewe W, Crommen J, Hubert P (2003) An analysis of the SFSTP guide on validation of chromatographic bioanalytical methods: progress and limitations. J Pharm Biomed Anal 32: 753–765 12899965 10.1016/s0731-7085(03)00182-1

[bib2] Boulanger B, Rozet E, Moonen F, Rudaz S, Hubert P (2009) A risk-based analysis of the AAPS conference report on quantitative bioanalytical methods validation and implementation. J Chromatogr B Analyt Technol Biomed Life Sci 877: 2235–2243 10.1016/j.jchromb.2009.06.019 19577965

[bib3] Carden CP, Sarker D, Postel-Vinay S, Yap TA, Attard G, Banerji U, Garrett MD, Thomas GV, Workman P, Kaye SB, de Bono JS (2010) Can molecular biomarker-based patient selection in phase I trials accelerate anticancer drug development? Drug Discov Today 15: 88–97 19961955 10.1016/j.drudis.2009.11.006

[bib4] Cummings J, Ranson M, Butt F, Moore D, Dive C (2007) Qualification of M30 and M65 ELISAs as surrogate biomarkers of cell death: long term antigen stability in cancer patient plasma. Cancer Chemother Pharmacol 60: 921–924 17333190 10.1007/s00280-007-0437-4

[bib5] Cummings J, Ward TH, Greystoke A, Ranson M, Dive C (2008) Biomarker method validation in anticancer drug development. Br J Pharmacol 153: 646–656 17876307 10.1038/sj.bjp.0707441 PMC2259203

[bib6] Dean E, Jodrell D, Connolly K, Danson S, Jolivet J, Durkin J, Morris S, Jowle D, Ward T, Cummings J, Dickinson G, Aarons L, Lacasse E, Robson L, Dive C, Ranson M (2009) Phase I trial of AEG35156 administered as a 7-day and 3-day continuous intravenous infusion in patients with advanced refractory cancer. J Clin Oncol 27: 1660–1666 19237630 10.1200/JCO.2008.19.5677

[bib7] DeSilva B, Smith W, Weiner R, Kelley M, Smolec J, Lee B, Khan M, Tacey R, Hill H, Celniker A (2003) Recommendations for the bioanalytical method validation of ligand-binding assays to support pharmacokinetic assessments of macromolecules. Pharm Res 20: 1885–1900 14661937 10.1023/b:pham.0000003390.51761.3d

[bib8] Fast DM, Kelley M, Viswanathan CT, O'Shaughnessy J, King SP, Chaudhary A, Weiner R, DeStefano AJ, Tang D (2009) Workshop report and follow-up--AAPS Workshop on current topics in GLP bioanalysis: Assay reproducibility for incurred samples--implications of Crystal City recommendations. AAPS J 11: 238–241 19381839 10.1208/s12248-009-9100-9 PMC2691460

[bib9] Feinberg M (2007) Validation of analytical methods based on accuracy profiles. J Chromatogr A 1158: 174–183 17343863 10.1016/j.chroma.2007.02.021

[bib10] Feinberg M, Boulanger B, Dewe W, Hubert P (2004) New advances in method validation and measurement uncertainty aimed at improving the quality of chemical data. Anal Bioanal Chem 380: 502–514 15365679 10.1007/s00216-004-2791-y

[bib11] Findlay JW (2008) Specificity and accuracy data for ligand-binding assays for macromolecules should be interpreted with caution. AAPS J 10: 433–434 18633714 10.1208/s12248-008-9047-2 PMC2761695

[bib12] Findlay JW (2009) Some important considerations for validation of ligand-binding assays. J Chromatogr B Analyt Technol Biomed Life Sci 877: 2191–2197 10.1016/j.jchromb.2008.10.045 19022709

[bib13] Findlay JW, Smith WC, Lee JW, Nordblom GD, Das I, DeSilva BS, Khan MN, Bowsher RR (2000) Validation of immunoassays for bioanalysis: a pharmaceutical industry perspective. J Pharm Biomed Anal 21: 1249–1273 10708409 10.1016/s0731-7085(99)00244-7

[bib14] Greystoke A, Cummings J, Ward TH, Simpson K, Renehan A, Butt F, Moore D, Gietema J, Blackhall F, Ranson M, Hughes A, Dive C (2008) Optimisation of circulating biomarkers of cell death for routine clinical use. Ann Oncol 19: 990–995 18304966 10.1093/annonc/mdn014

[bib15] Hou JM, Greystoke A, Lancashire L, Cummings J, Ward T, Board R, Amir E, Hughes S, Krebs M, Hughes A, Ranson M, Lorigan P, Dive C, Blackhall FH (2009) Evaluation of circulating tumor cells and serological cell death biomarkers in small cell lung cancer patients undergoing chemotherapy. Am J Pathol 175: 808–816 19628770 10.2353/ajpath.2009.090078 PMC2716975

[bib16] Jain RK, Duda DG, Willett CG, Sahani DV, Zhu AX, Loeffler JS, Batchelor TT, Sorensen AG (2009) Biomarkers of response and resistance to antiangiogenic therapy. Nat Rev Clin Oncol 6: 327–338 19483739 10.1038/nrclinonc.2009.63 PMC3057433

[bib17] Kelley M, DeSilva B (2007) Key elements of bioanalytical method validation for macromolecules. AAPS J 9: E156–E163 17614356 10.1208/aapsj0902017 PMC2751404

[bib18] Lee JW, Devanarayan V, Barrett YC, Weiner R, Allinson J, Fountain S, Keller S, Weinryb I, Green M, Duan L, Rogers JA, Millham R, O'Brien PJ, Sailstad J, Khan M, Ray C, Wagner JA (2006) Fit-for-purpose method development and validation for successful biomarker measurement. Pharm Res 23: 312–328 16397743 10.1007/s11095-005-9045-3

[bib19] Lee JW, Figeys D, Vasilescu J (2007) Biomarker assay translation from discovery to clinical studies in cancer drug development: quantification of emerging protein biomarkers. Adv Cancer Res 96: 269–298 17161683 10.1016/S0065-230X(06)96010-2

[bib20] Lee JW, Weiner RS, Sailstad JM, Bowsher RR, Knuth DW, O'Brien PJ, Fourcroy JL, Dixit R, Pandite L, Pietrusko RG, Soares HD, Quarmby V, Vesterqvist OL, Potter DM, Witliff JL, Fritche HA, O'Leary T, Perlee L, Kadam S, Wagner JA (2005) Method validation and measurement of biomarkers in nonclinical and clinical samples in drug development: a conference report. Pharm Res 22: 499–511 15846456 10.1007/s11095-005-2495-9

[bib21] Ludwig JA, Weinstein JN (2005) Biomarkers in cancer staging, prognosis and treatment selection. Nat Cancer Rev 5: 845–856 10.1038/nrc1739 16239904

[bib22] Mahler HC, Friess W, Grauschopf U, Kiese S (2009) Protein aggregation: pathways, induction factors and analysis. J Pharm Sci 98: 2909–2934 18823031 10.1002/jps.21566

[bib23] McShane LM, Hunsberger S, Adjei AA (2009) Effective incorporation of biomarkers into phase II trials. Clin Cancer Res 15: 1898–1905 19276274 10.1158/1078-0432.CCR-08-2033 PMC2874890

[bib24] Nowatzke W, Wood E (2007) Best practices during bioanalytical method validation for the characterisation of assay reagents and the evaluation of analyte stability in assay standards, quality controls and study samples. AAPS J 9: E117–E122 17614353 10.1208/aapsj0902013 PMC2751400

[bib25] Rozet E, Hubert C, Ceccato A, Dewe W, Ziemons E, Moonen F, Michail K, Wintersteiger R, Streel B, Boulanger B, Hubert P (2007) Using tolerance intervals in pre-study validation of analytical methods to predict in-study results. The fit-for-future-purpose concept. J Chromatogr A 1158: 126–137 17420025 10.1016/j.chroma.2007.03.102

[bib26] Sarker D, Workman P (2007) Pharmacodynamic biomarkers for molecular cancer therapeutics. Adv Cancer Res 96: 213–268 17161682 10.1016/S0065-230X(06)96008-4

[bib27] Shah VP, Midha KK, Dighe S, McGilveray IJ, Skelly JP, Yacobi A, Layloff T, Viswanathan CT, Cook CE, McDowall RD (1991) Analytical methods validation: bioavailability, bioequivalence and pharmacokinetic studies. Conference report. Eur J Drug Metab Pharmacokinet 16: 249–255 1823867 10.1007/BF03189968

[bib28] Shah VP, Midha KK, Findlay JW, Hill HM, Hulse JD, McGilveray IJ, McKay G, Miller KJ, Patnaik RN, Powell ML, Tonelli A, Viswanathan CT, Yacobi A (2000) Bioanalytical method validation--a revisit with a decade of progress. Pharm Res 17: 1551–1557 11303967 10.1023/a:1007669411738

[bib29] Tan DS, Thomas GV, Garrett MD, Banerji U, de Bono JS, Kaye SB, Workman P (2009) Biomarker-driven early clinical trials in oncology: a paradigm shift in drug development. Cancer J 15: 406–420 19826361 10.1097/PPO.0b013e3181bd0445

[bib30] Viswanathan CT, Bansal S, Booth B, Destefano AJ, Rose MJ, Sailstad J, Shah VP, Skelly JP, Swann PG, Weiner R (2007) Quantitative bioanalytical methods validation and implementation: best practices for chromatographic and ligand binding assays. Pharm Res 9: E30–E42 10.1007/s11095-007-9291-7 17458684

